# Supplementary material for: Batryticatus Bombyx Protects Dopaminergic Neurons against MPTP-Induced Neurotoxicity by Inhibiting Oxidative Damage
Source: Antioxidants (Basel). 2019 Nov 21;8(12):574. doi: 10.3390/antiox8120574 (PMC6943627; doi:10.3390/antiox8120574)
Supplement: Supplementary file 1 [file antioxidants-08-00574-s001.pdf]

Supplemental Data

Batryticatus Bombyx Protects Dopaminergic Neurons Against MPTP-Induced Neurotoxicity by Inhibiting Oxidative Damage

Hye-Sun Lim, Joong-Sun Kim, Byeong Cheol Moon, Seung Mok Ryu, Jun Lee and Gunhyuk Park *

Herbal Medicine Resources Research Center, Korea Institute of Oriental Medicine, 111 Geonjae-ro, Naju-si, Jeollanam-do, 58245, Korea

* Correspondence: gpark@kiom.re.kr or parkgunhyuk@gmail.com; Tel.: +82-61-338-7112

Materials and Methods

Brain Tissue Preparation

On day 7 after MPTP treatment, mice were anesthetized immediately and perfused transcardially with 0.05 M phosphate-buffered saline (PBS; Sigma-Aldrich), followed by addition of cold 4% paraformaldehyde (PFA; Sigma-Aldrich) in 0.1 M phosphate buffer. Brains were removed and post-fixed in 0.1 M phosphate buffer (Sigma-Aldrich) containing 4% PFA overnight at 4°C, and immersed in a solution containing 30% sucrose in 0.05 M PBS for cryoprotection. Serial 30-μm-thick coronal sections were cut on a freezing microtome (Leica Instruments GmbH, Nussloch, Germany) and stored in a cryoprotectant (25% ethylene glycol; Sigma-Aldrich), 25% glycerol (Sigma-Aldrich), and 0.05 M phosphate buffer at 4°C until use.

Immunohistochemistry (IHC) Analysis

Brain sections were rinsed briefly in PBS, treated with 1% hydrogen peroxide (Sigma-Aldrich) for 15 min, and incubated with rabbit anti-tyrosine hydroxylase (TH) (1:1000) overnight at 4 °C in the presence of 0.3% Triton X-100 (Vector Laboratories, Burlingame, CA, USA) and normal goat serum (Vector Laboratories). After rinsing in PBS, the sections were incubated with biotinylated anti-rabbit IgG (Vector Laboratories) (1:200) for 90 min, rinsed, and incubated with ABC (Vector Laboratories) (1:100) for 1 h at room temperature. Peroxidase activity was visualized with DAB (Sigma-Aldrich) in 0.05 M Tris-buffered saline (Sigma-Aldrich). After several rinses with PBS, the specimens were mounted on gelatin-coated slides, dehydrated, and cover-slipped using histo-mount medium. Images were photographed at 40× and 100× magnification using an optical light microscope (Olympus Microscope System BX53; Olympus, Tokyo, Japan) equipped with a 20× objective lens.

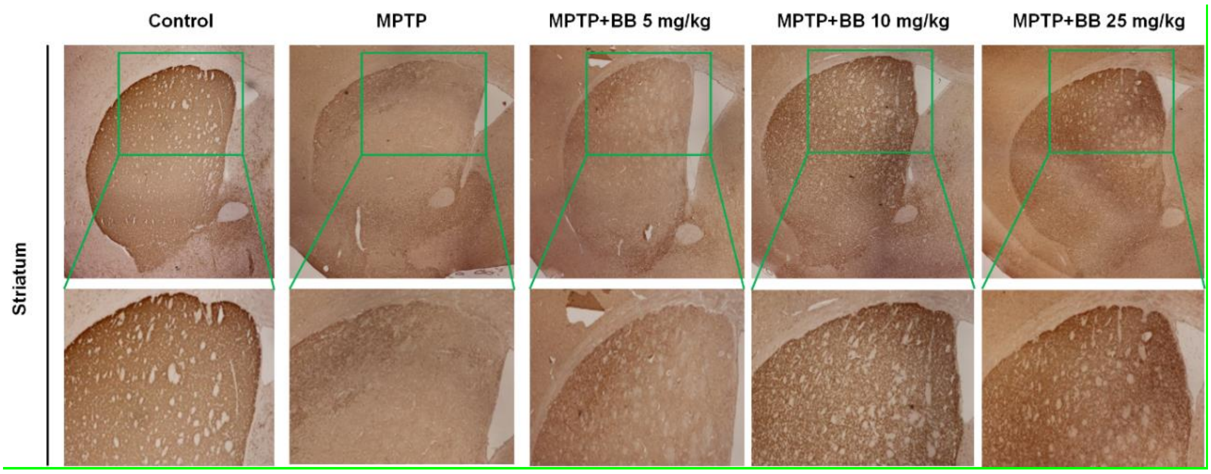

Figure S1. Effects of Batryticatus Bombyx (BB) on MPTP-induced dopaminergic neuronal death. Dopaminergic neurons were visualized by TH-specific immunostaining. Representative photomicrographs of the striatum (ST) were taken.
